# An Unusual Presentation of Congenital Esophageal Stenosis Due to Tracheobronchial Remnants in a Newborn Prenatally Diagnosed with Duodenal Atresia

**DOI:** 10.5334/jbr-btr.881

**Published:** 2015-12-30

**Authors:** Cindy Mai, Luc Breysem, Gert De Hertogh, Dirk Van Raemdonck, Maria-Helena Smet

**Affiliations:** 1 University Hospitals Gasthuisberg, Leuven, BE

**Keywords:** Esophageal stenosis, Constriction, Duodenal obstruction, Infant

## Abstract

Congenital esophageal stenosis due to tracheobronchial remnants is defined as an intrinsic stenosis of the esophagus caused by congenital architectural abnormalities of the esophageal wall. Although CES is present at birth, it remains asymptomatic till at the age of 4–10 months, when solid food is introduced. Here we present a case diagnosed in the neonatal period after urgent cesarean for an associated duodenal atresia complicated with perforation. There is a mutual association between duodenal atresia and congenital esophageal stenosis. When duodenal atresia is diagnosed, think of possible associated esophageal abnormalities, especially when duodenal atresia is complicated by gastric perforation prenatally.

## Introduction

Congenital esophageal stenosis due to tracheobronchial remnants (CES-TBR) is defined as an intrinsic stenosis of the esophagus caused by congenital architectural abnormalities of the esophageal wall [1]. These abnormalities in the esophageal wall are assumed to be caused by an error in the separation of the foregut into the trachea and the esophagus [2,3], as in esophageal atresia with or without tracheoesophageal fistula. Esophageal atresia is the most frequent abnormality associated with CES-TBR, which strongly suggests a common origin of disease. CES-TBR is rarely diagnosed in the neonatal period, unless there is an associated esophageal atresia. In the literature, most cases present at the age of 4–10 months when solid food is introduced [4]; however, mild cases can stay unnoticed until adulthood. These patients typically have a lifelong history of mild dysphagia and recurrent food impactions [5]. Here we present a case diagnosed in the neonatal period after urgent cesarean for an associated duodenal atresia complicated with perforation.

## Case report

**Figure 1:**
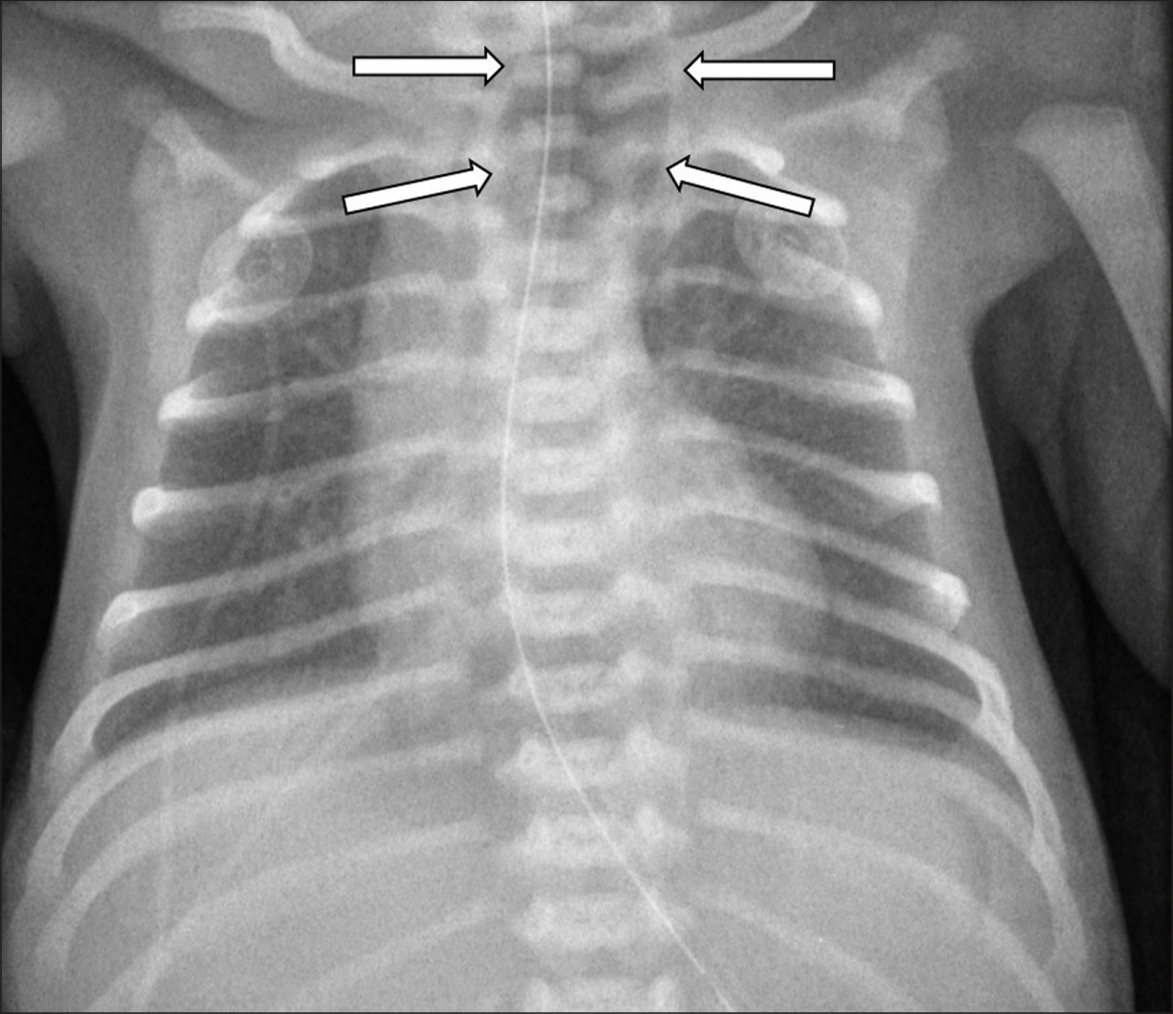
Thoracic X-ray shows a progressive widening air column (white arrows) projecting on the cervicothoracic region.

**Figure 2:**
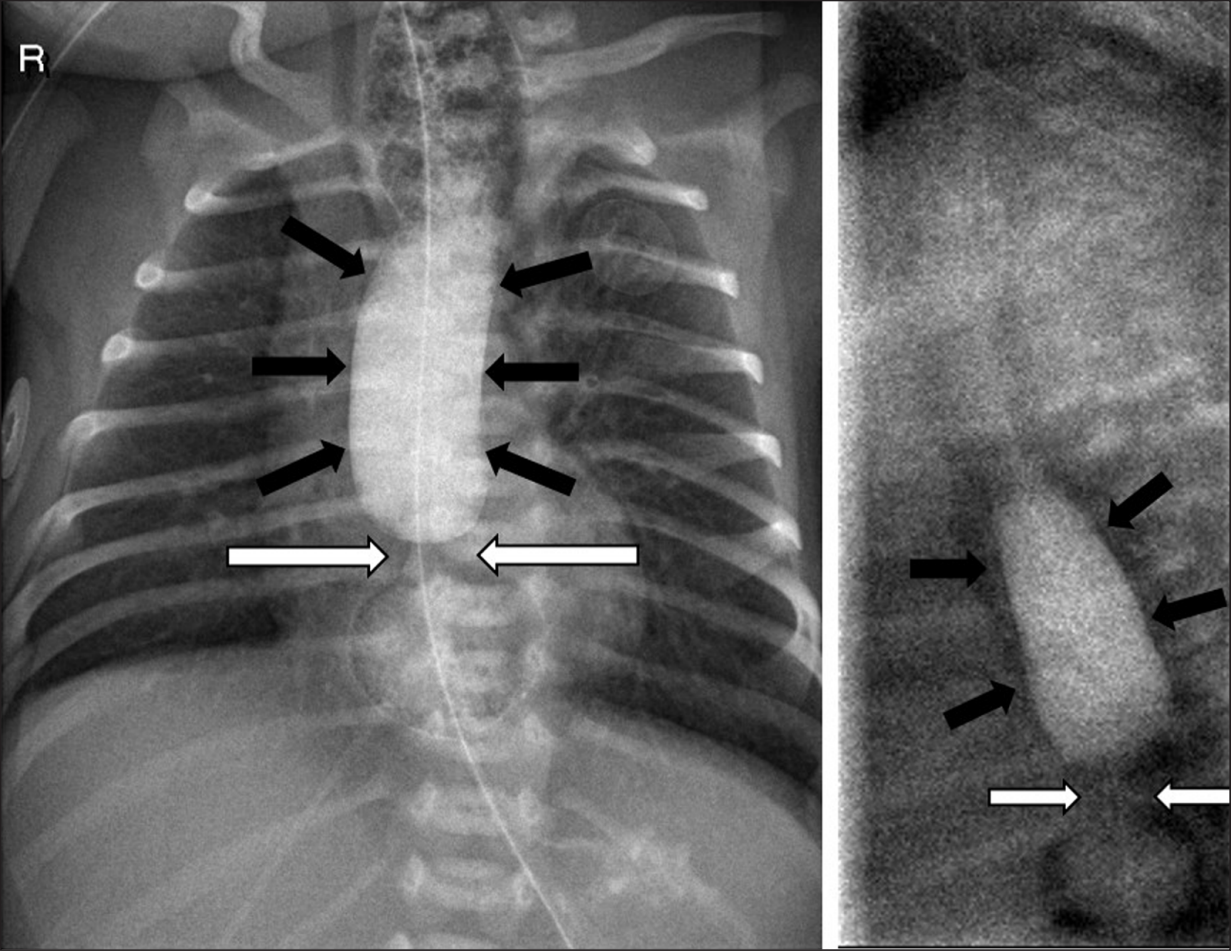
Contrast study (AP and profile) reveals a dilated esophagus (black arrows) with stenosis at the transition from the middle to the distal third (white arrows). There is air and contrast passage in the distal part of the esophagus, past the stenosis.

**Figure 3:**
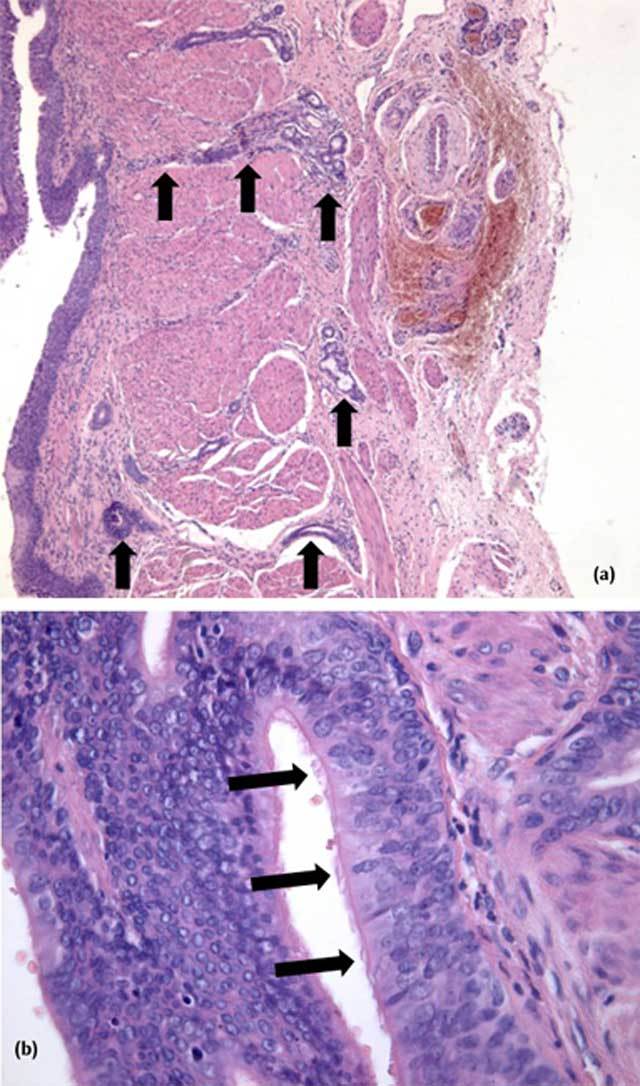
(a) Longitudinal slide, Hematoxylin and Eosin (HE) staining, 50x magnification: Abnormal glands (black arrows) in the submucosa reaching through the muscularis propria almost to the adventitia. (b) HE staining, 400x magnification: Pseudostratified columnar epithelium with cilia (black arrows).

An infant prenatally diagnosed with duodenal atresia was delivered at 33 weeks by urgent cesarean because of suspected gastrointestinal perforation. Postnatal abdominal X-ray showed the typical double-bubble sign of duodenal atresia and an ultrasound confirmed the presence of free fluid suggestive of perforation. During surgery a perforation of the posterior wall of the gastric antrum was found, and closed. Soon afterwards signs of respiratory distress and hypersalivation emerged. On serial thoracic X-ray, a progressive widening air column was seen projecting on the cervicothoracic area (Figure 1). A contrast study showed opacification of a dilated esophagus, with constriction at the lower esophagus (Figure 2). Air is also seen in the distal part of the esophagus past the site of constriction. The stricture was excised with repair by end-to-end anastomosis. Histopathology of the specimen showed the presence of a respiratory-type mucosa, – ciliated pseudostratified columnar epithelium – and bronchial-type glands in the submucosa extending right to the adventitia (Figure 3).

## Discussion

Congenital esophageal stenosis (CES) due to tracheobronchial remnants was already defined in 1987 as an intrinsic stenosis of the esophagus due to congenital architectural abnormalities of the esophageal wall [1]. There are three types of CES, each with distinguishing characteristics. The first is characterized by the presence of ectopic tracheobronchial tissue (e.g., ciliated epithelium) and respiratory mucous gland and/or cartilage. Type 2 consists of a membranous diaphragm and type 3 of a fibromuscular hypertrophy. Our case was a type 1, which is the most frequent type.

The stenosis is typically situated at the distal or lower end of the esophagus. The incidence of CES is estimated to be 1 in 25,000 to 50,000 live births. There is no gender predilection, although some studies showed a slightly male predominance [6].

Although CES is present at birth, it is seldom diagnosed in the neonatal period. In most cases, symptoms occur between the ages of 4–10 months, when solid food is introduced. The major symptoms in this age group are dysphagia and regurgitation. Other symptoms include saliva excess, failure to thrive and recurrent respiratory infections due to aspiration [4,5,6].

CES can be an isolated finding or may be associated with other congenital anomalies, most frequently with esophageal atresia – with or without tracheoesophageal fistula [6,7]. An association with duodenal atresia, like in our case, is also seen but less frequent [6]. In these cases, the stomach is isolated from the gastrointestinal tract with an accumulation of gastric fluid as a consequence. Thus, duodenal atresia is likely to give a more pronounced double-bubble image on a prenatal ultrasound and free fluid when it is complicated with perforation. Therefore, if duodenal atresia is complicated by gastrointestinal perforation, focus on CES or other causes of mechanical obstruction proximal of the stomach.

Endoscopic dilatation is performed in most cases of CES, although the results are only temporary, with rapid reoccurrence of the symptoms and the need for repeated dilatations. Furthermore, there is also a risk of perforation. The preferred treatment for CES is a limited excision of the stenotic segment with end-to-end anastomosis. Limited resection of the stenosis gives good long-term results and postoperative complications are rare. Anastomotic stenosis is the most frequent complication and can be treated with endoscopic dilatation [6].

## Competing Interests

The authors declare that they have no competing interests.
